# A systematic review of antimicrobial resistance among World Health Organization Bacterial Priority Pathogens in Syria (2015-2025)

**DOI:** 10.1016/j.ijregi.2026.100980

**Published:** 2026-08-18

**Authors:** Bayan Galal, Nadia Barouk, Ahmad Kejah, Marwan Osman, Nabil Karah, Salam Abbara, Yasir Farrouh, Shaffi Fazaludeen Koya, Antoine Abou Fayad, Souha S. Kanj, Ahmad Darwish, Omar Dewachi, Aula Abbara

**Affiliations:** 1 Department of Medicine, Perelman School of Medicine, University of Pennsylvania, Philadelphia, USA; 2 Department of Medicine, Rowan-Virtua School of Osteopathic Medicine, Stratford, USA; 3 Department of Microbiology, Aleppo University, Aleppo, Syria; 4 Department of Neurosurgery, Yale University School of Medicine, New Haven, Connecticut, USA; 5 Department of Molecular Biology, Umea University, Umea, Sweden; 6 Epidemiology and Modelling of Antibiotic Evasion Unit, Pasteur Institute, Paris-Cité University, Paris, France; 7 Department of Infectious Diseases, Syrian Ministry of Health, Damascus, Syria; 8 Antimcirobial Resistance, WHO Regional Office for the Eastern Mediterranean, Cairo, Egypt; 9 Department of Microbiology, American University of Beirut, Beirut, Lebanon; 10 Department of Medicine, and Center for Infectious Diseases research, Faculty of Medicine, American University of Beirut, Beirut, Lebanon; 11 Private Microbiology Laboratory, Damascus, Syria; 12 Department of Anthropology, Rutgers University, New Brunswick, USA; 13 Department of Infectious Diseases, Imperial College London, London, UK; 14 Syria Public Health Network, London, UK; 15 Yale Institute for Global Health, New Haven, Connecticut, USA

**Keywords:** Antimicrobial resistance, Syria, Systematic review, Surveillance, Conflict-affected setting

## Abstract

**Objectives:**

Antimicrobial resistance (AMR) is an urgent public health threat, but empirical data from Syria remain limited after prolonged conflict-related disruption of health care, laboratory, and surveillance systems. We aimed to synthesize the available evidence on AMR in Syria.

**Methods:**

We searched PubMed, EMBASE, and Google Scholar for English- or Arabic-language studies published from 2015 to 2025 that reported AMR among organisms included in the World Health Organization Bacterial Priority Pathogen List organisms clinical or screening samples collected in Syria. Two reviewers independently screened records and extracted study, specimen, organism, susceptibility testing, and resistance data. Findings were synthesized narratively and summarized using study-level medians and interquartile ranges (IQRs).

**Results:**

Of 203 records identified, 22 studies were included, representing 6367 samples. Studies were largely hospital-based and concentrated in Damascus, Aleppo, and Lattakia. Third-generation cephalosporin resistance was high among *Enterobacterales*, including *Escherichia coli* (83.6%; IQR 71.2-91.6), *Klebsiella pneumoniae* (96.3%; IQR 80.5-100.0), and *Enterobacter* spp. (90.5%; IQR 56.3-93.5). Median carbapenem resistance in *Acinetobacter baumannii* was 79.4% (IQR 51.3-91.7). Fluoroquinolone resistance was common across Gram-negative pathogens. Methicillin resistance among *Staphylococcus aureus* was 70.5% (IQR 68.2-72.8).

**Conclusion:**

Syrian AMR data remain sparse and geographically uneven, but the available evidence suggests substantial resistance. Standardized national surveillance, microbiology capacity, local antibiograms, and context-specific empiric treatment guidelines are urgently needed.

## Highlights

• Data on 6367 bacterial samples from Syria were analyzed.
• Third-generation cephalosporin resistance was high among Gram-negative bacteria.
• Median carbapenem resistance was 79% for *A. baumannii*.
• Published data are concentrated in major Syrian cities and hospitals.
• Antimicrobial resistance surveillance for key pathogens must be implemented.

## Introduction

Multiple drivers have been identified as contributing to the emergence and spread of antimicrobial resistance (AMR) [1,2]. In Syria, almost 14 years of conflict devastated the health system with widespread destruction of health care facilities, including many clinical and surveillance laboratories [3]. Consequently, while a clinical consensus among health care workers suggests high AMR prevalence, empirical data to characterize resistance patterns and optimal treatment pathways remain sparse [4]. This also limits the contribution of AMR epidemiology in Syria to regional and global surveillance [5]. In 2021, it was estimated that 23,000 people died of sepsis in Syria, with approximately 6,000 deaths associated with AMR; although this is likely a significant undercount of the true burden, it is indicative of the associated mortality [6].

A 2018 scoping review of Syrian AMR data from before and during the conflict suggested that the available data were sparse and of poor quality [7]. Since then, several publications have described resistance patterns in individual organisms, but these data have not been systematically synthesized. This review aims to characterize the available evidence on AMR among organisms included in the World Health Organization (WHO) Bacterial Pathogen Priority List (BPPL), which were selected because of their public health importance and resistance burden, in Syria, to inform national clinical guidelines, strengthen diagnostic laboratory prioritization, and support surveillance [8].

## Methods

### Search strategy

A systematic literature search was conducted in June 2025, following the Preferred Reporting Items for Systematic Reviews and Meta-Analyses (PRISMA) guidelines (Table S1). The review was not registered. An unpublished internal protocol was prepared before screening. PubMed, EMBASE, and Google Scholar were searched for studies published between January 2015 and June 2025. Citation searching of included studies was also performed. Search terms combined “Syria” or “Syrian” with AMR descriptors (“antibiotic resistance,” “multidrug resistant,” “ESBL (extended-spectrum beta-lactamases),” “carbapenem-resistant,” etc.) and pathogen names from the 2024 WHO BPPL, including *Acinetobacter baumannii*; Enterobacterales (*Escherichia coli, Klebsiella pneumoniae, Enterobacter* spp., *Serratia* spp., *Proteus* spp., *Citrobacter* spp., and *Morganella* spp.); *Pseudomonas aeruginosa; Staphylococcus aureus* (including methicillin-resistant *S. aureus* [MRSA]); *Enterococcus faecium* (including vancomycin-resistant enterococci); *Neisseria gonorrhoeae; Salmonella Typhi*; non-typhoidal *Salmonella; Shigella* spp.; Group A *Streptococcus*; Group B *Streptococcus; Streptococcus pneumoniae; Haemophilus influenzae*; and *Mycobacterium tuberculosis*. The full search strategy is provided in Tables S2 and S3.

### Eligibility and exclusion criteria

We included studies that reported on BPPL pathogens isolated from clinical or screening samples from Syrians residing inside Syria between 2015 and 2025 [8]. Eligible study types comprised peer-reviewed primary research, hospital antibiograms, surveillance reports, non-governmental organization or institutional gray literature, and systematic reviews. Case reports were eligible only if they included at least 10 patients. Publications were limited to those in English or Arabic. Reviews, commentaries, conference abstracts, or other non-original sources without primary data were excluded. Studies reporting on populations outside Syria or reporting only animal, environmental, or *in vitro* isolates were also excluded, as were records for which the full text could not be obtained.

### Study selection

Search results were imported into Rayyan, where duplicates were removed. Two reviewers (BG and NB) independently screened all titles and abstracts, followed by full-text review of potentially eligible studies. Any disagreements were resolved by discussion or, when necessary, by adjudication by a third reviewer (AA).

### Data extraction

Data were extracted into a Microsoft Excel sheet. One reviewer per study collected information on bibliographic details, study characteristics (design, setting, and population), sample information (facility type and specimen type), and pathogen-resistance findings. Where available, antimicrobial susceptibility testing (AST) methods, antibiotics tested, and resistance rates were also recorded. AMR was defined according to the susceptibility interpretation reported by each included study. Resistance classifications were extracted as reported by the original investigators using their specified breakpoint criteria and were not reclassified using a uniform set of breakpoints. Any uncertainties in data extraction were referred to a third reviewer. To allow both organism-specific and study-level synthesis, data for *K. pneumoniae, E. coli, Enterobacter* spp.*, P. aeruginosa, A. baumannii,* and *S. aureus* were extracted into dedicated worksheets, while general metadata were captured in a master sheet.

When studies reported resistance percentages and denominators but not raw counts, the number of resistant isolates was reconstructed by multiplying the percentage by the denominator and rounding to the nearest integer. We also noted instances where rounding ambiguity prevented reliable reconstruction.

### Data synthesis

Extracted data were synthesized narratively and in summary tables, stratified by pathogen and AMR phenotype (including organism-specific susceptibility/resistance patterns and multidrug resistance, where reported). Given substantial heterogeneity in study design, populations, specimen types, laboratory methods, and antimicrobial testing panels, a formal meta-analysis was not performed.

### Data analysis

Resistance data were summarized using a two-stage approach to reduce the influence of variable antibiotic panels across studies. First, within each study, organism-specific resistance for a given antibiotic class was summarized as the median resistance proportion across individual antibiotics tested within that class. Second, these study-level class-specific estimates were aggregated across studies using the median and interquartile range (IQR), such that each study contributed one observation per isolate-antibiotic class combination. This approach was chosen to provide robust descriptive summaries in the setting of heterogeneous reporting and to avoid over-weighting studies that tested a larger number of antibiotics within the same class. Statistical analyses, as well as select table and figure generation, were conducted using R version 4.5.2 [9] in RStudio version 2025.09.2+418 [10].

### Quality assessment

We assessed the methodological quality (risk of bias) of included studies using the National Heart, Lung, and Blood Institute (NHLBI) Quality Assessment Tool for Observational Cohort and Cross-Sectional Studies (14-item checklist), applying the accompanying guidance for interpretation of each item. Each item was coded as Yes, No, Cannot Determine, or Not Applicable, in accordance with NHLBI guidance. An overall quality rating (Good/Fair/Poor) was assigned based on a qualitative judgment of internal validity and risk of bias, with emphasis on potential selection bias, measurement bias, information bias, and confounding.

## Results

### Study selection

**Figure 1:**
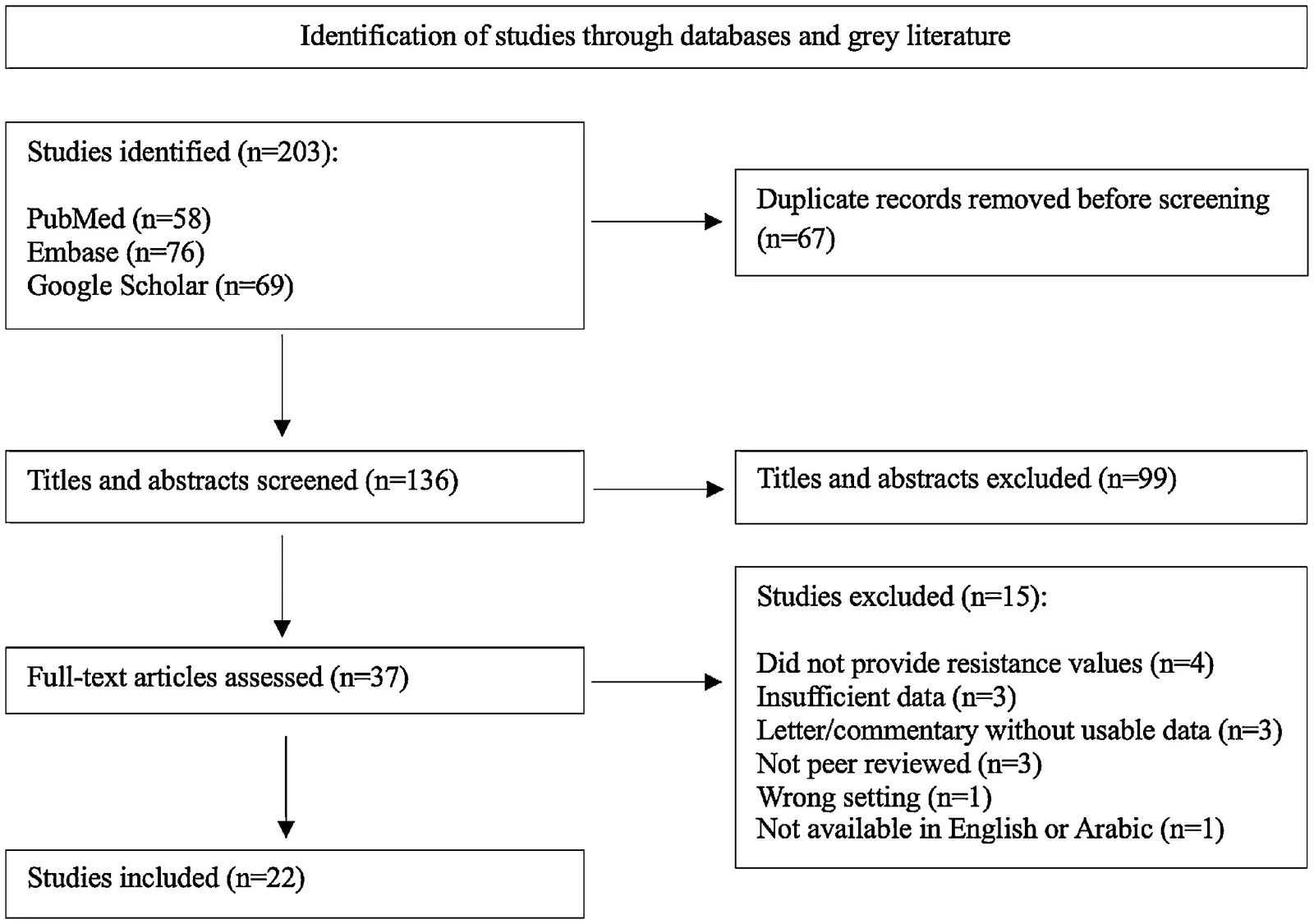
PRISMA flow diagram of study selection. Flow diagram showing the identification, screening, eligibility assessment, and final inclusion of studies in the review. PRISMA, Preferred Reporting Items for Systematic Reviews and Meta-Analyses.

In total, 203 records were identified (PubMed n = 58, EMBASE n = 76, Google Scholar n = 69). After 67 duplicates were removed, 136 records underwent title and abstract screening. Thirty-seven studies were assessed in full text, of which 22 met the eligibility criteria and were included. The selection process is illustrated in the PRISMA flow diagram (Figure 1).

### Study characteristics

A total of 22 studies were included, representing 6,367 samples overall. The median sample size was 111.5 isolates per study (IQR 70.25-182.25). Study characteristics are summarized in Table 1.

**Table 1:** Summary characteristics of included studies.

Study (last name, year)	Years of data	City	Setting	Sample type	Sample size	Organisms reported	AST method used	Quality assessment
Alheib et al. [11]	2010-2011	Aleppo	Inpatient	Not reported	Total patients/isolates: 123; culture-positive: 123	*K. pneumoniae* (n = 24)*; E. coli* (n = 99)	Broth microdilution following CLSI guidelines	Fair
Al-Subol et al. [12]	2010-2012	Aleppo	Inpatient	Not reported	Total patients/isolates:199; culture-positive: 199	*K. pneumoniae* (n = 40)*; E. coli* (n = 159)	Kirby-Bauer following CLSI 2010 guidelines	Fair
Mahfoud et al. [13]	2011-2012	Aleppo	ICU (n = 138); inpatient (n = 39)	Respiratory (n = 138); urine (n = 39)	Total patients/isolates: not reported; culture-positive: 177	*P. aeruginosa* (n = 177)	Kirby-Bauer following CLSI guidelines	Fair
Shbib et al. [14]	2012-2013	Damascus	Not reported	Urine	Total patients/isolates: 75; culture-positive: 65	*E. coli* (n = 65)	E-test on Mueller-Hinton agar; no CLSI/EUCAST standard reported	Fair
Ahmad et al. [15]	2010-2011	Damascus	Inpatient	Wound	Total patients/isolates: 100; culture-positive: 48	*P. aeruginosa* (n = 18)	Kirby-Bauer disk diffusion on Mueller-Hinton agar; no CLSI/EUCAST standard reported	Fair
Omran et al. [16]	2015	Damascus	Not reported	Urine (n = 195); pus (n = 90); sputum (n = 37); wound (n = 81); other (n = 54)	Total patients/isolates: 457; culture-positive: 457	*E. coli* (n = 224)*; P. aeruginosa* (n = 106); *S. aureus* (n = 129)	Kirby-Bauer disk diffusion; no CLSI/EUCAST standard reported	Fair
Ahmad [17]	2015-2016	Damascus	Inpatient	Wound	Total patients/isolates: 466; culture-positive: 100	*K. pneumoniae* (n = 8)*; E. coli* (n = 14)*; Enterobacter* spp. (n = 3)*; P. aeruginosa* (n = 22)	Kirby-Bauer disk diffusion; Mueller-Hinton agar; no CLSI/EUCAST standard reported	Fair
Yazigi et al. [18]	2015-2016	Lattakia	Inpatient (n = 170); ICU (n = 30)	Urine	Total patients/isolates: 200; culture-positive: 40	*K. pneumoniae* (n = 4)*; E. coli* (n = 16)*; P. aeruginosa* (n = 4)	Mueller-Hinton agar susceptibility testing; no CLSI/EUCAST standard reported	Fair
Daoud et al. [19]	2018-2019	Damascus	Inpatient	Wound	Total patients/isolates: 51; culture-positive: 51	*E. coli* (n = 51)	Kirby-Bauer disk diffusion method on Mueller-Hinton agar, interpreted using CLSI criteria, 2018	Fair
Karamya et al. [20]	2016-2018	Damascus, Homs, Lattakia, Tartous	Not reported	Blood; sputum; urine; wound (counts not reported)	Total patients/isolates: not reported; culture-positive: 3,577	*K. pneumoniae; E. coli; Enterobacter* spp.*; P. aeruginosa; Acinetobacter baumannii; S. aureus* (counts not reported)	Kirby-Bauer following CLSI guidelines	Fair
Al Abbas et al. [21]	2020	Homs	Inpatient	Wound	Total patients/isolates: 65; culture-positive: 63	*S. aureus* (n = 26); *P. aeruginosa* (n = 12); *K. pneumoniae* (n = 7); *E. coli* (n = 5); *Enterobacter* spp. (n = 4); *A. baumannii* (n = 4)	Kirby-Bauer following CLSI guidelines	Fair
Harfouch et al. [22]	2021-2022	Lattakia	Inpatient	Wounds; urine; body fluids; endotracheal aspirations (counts not reported)	Total patients/isolates: not reported; culture-positive:100	*P. aeruginosa* (n = 100)	Kirby-Bauer on Mueller-Hinton agar; no CLSI/EUCAST standard reported	Fair
Karah et al. [23]	2020-2021	Idlib	Outpatient	Urine	Total patients/isolates: not reported; culture-positive: 74	*E. coli* (n = 61)*, K. pneumoniae* (n = 12)	Disk diffusion following EUCAST guidelines	Fair
Alyousef et al. [24]	2021-2022	Damascus	Inpatient	Pus (n = 57); Blood (n = 14); Urine (n = 14), Sputum (n = 10); Other (n = 5)	Total patients/isolates: not reported; culture-positive: 100	*S. aureus* (n = 100)	Cefoxitin disk diffusion, CLSI; mecA PCR for MRSA confirmation	Fair
Arab et al. [25]	2021-2022	Aleppo	Outpatient	Sputum (n = 68); Bronchial washings (n = 182); Transtracheal aspirations (n = 18)	Total patients/isolates: 268; culture-positive: 150; bacterial isolates analyzed: 136	*K. pneumoniae* (n = 8)*; E. coli* (n = 12)*; P. aeruginosa* (n = 34)*; A. baumannii* (n = 38); *S. aureus* (n = 16)	Broth microdilution per NCCLS/CLSI Performance Standards, M100S Supplement, 26th edition, 2016	Fair
Atrash et al. [26]	2021-2022	Lattakia	Outpatient; inpatient (counts not reported)	Urine	Total patients/isolates: 386; culture-positive: 184	*E. coli; Enterobacter* spp.*; P. aeruginosa* (counts not reported)	Disk diffusion using Mueller-Hinton agar; no CLSI/EUCAST standard reported	Fair
Kahal et al. [27]	2020-2021	Damascus	Inpatient	Urine (n = 67); blood (n = 46); sputum (n = 41); skin (n = 12); CSF (n = 11)	Total patients/isolates: 116; culture-positive: 177	*E. coli* (n = 21)*; Enterobacter* spp. (n = 37)*; P. aeruginosa* (n = 27)*; S. aureus* (n = 58)	Kirby-Bauer on Mueller-Hinton agar following CLSI guidelines	Fair
Bourgi et al. [28]	2022-2023	Idlib	Inpatient	Urine (n = 105); sputum (n = 55); skin (n = 23); seminal fluid (n = 5); blood and CSF (n = 12)	Total patients/isolates: 300; patients who underwent antibiogram: 200; culture-positive: 185	*K. pneumoniae* (n = 37)*; E coli* (n = 60)*; Enterobacter* (n = 26)*; P. aeruginosa* (n = 7)*; S. aureus* (n = 23)	Kirby-Bauer disk diffusion on Mueller-Hinton agar; interpreted according to CLSI M39 guidelines	Fair
Al Debs et al. [29]	2023	Damascus	Inpatient	Wound	Total patients/isolates: 90; culture-positive: 30	*K. pneumoniae* (n = 30)	Kirby-Bauer disk diffusion method on Mueller-Hinton agar following CLSI guidelines	Fair
Jadeed et al. [30]	2019-2020	Damascus	Inpatient, ICU (counts not reported)	Blood (n = 46); urine (n = 31); CSF (n = 7); pus (n = 14); sputum (n = 26); peritoneal fluid (n = 1)	Total patients/isolates: 277; culture-positive: 125	*E. coli* (n = 54); *Klebsiella* spp. (n = 64); *Enterobacter* spp. (n = 3)	Disk diffusion was used to examine bacterial susceptibility. Results were interpreted according to BD charts. Some samples were tested using the BD Phoenix system	Fair
Khalil et al. [31]	2023-2024	Damascus	ICU	Endotracheal aspirate	Total patients/isolates: 105; culture-positive: 69	*K. pneumoniae* (n = 14)*; Enterobacter* spp. (n = 14)*; P. aeruginosa* (n = 5)*; A. baumannii* (n = 23)*; S. aureus* (n = 12)	Kirby-Bauer disk diffusion on Mueller-Hinton agar, interpreted using CLSI M100-S21 guidelines	Fair
Shanan et al. [32]	2021-2022	Aleppo	Inpatient	Not reported	Total patients/isolates: 430; culture-positive: 261	*P. aeruginosa* (n = 74)	Kirby-Bauer disk diffusion; selective media/culture-based identification; molecular confirmation by PCR for *P. aeruginosa* and resistance genes	Fair

Characteristics of the 22 studies included in the review, including publication year, study period, geographic location, clinical setting, specimen type, sample size, organisms reported, and antimicrobial susceptibility testing (AST) method used.

Abbreviations: *A. baumannii, Acinetobacter baumannii*; AST, antimicrobial susceptibility testing; BD, Becton Dickinson; CLSI, Clinical and Laboratory Standards Institute; CSF, cerebrospinal fluid; *E. coli, Escherichia coli*; EUCAST, European Committee on Antimicrobial Susceptibility Testing; ICU, intensive care unit; *K. pneumoniae, Klebsiella pneumoniae*; MRSA, methicillin-resistant *Staphylococcus aureus*; NCCLS, National Committee for Clinical Laboratory Standards; *P. aeruginosa, Pseudomonas aeruginosa*; PCR, polymerase chain reaction; *S. aureus, Staphylococcus aureus*.

**Figure 2:**
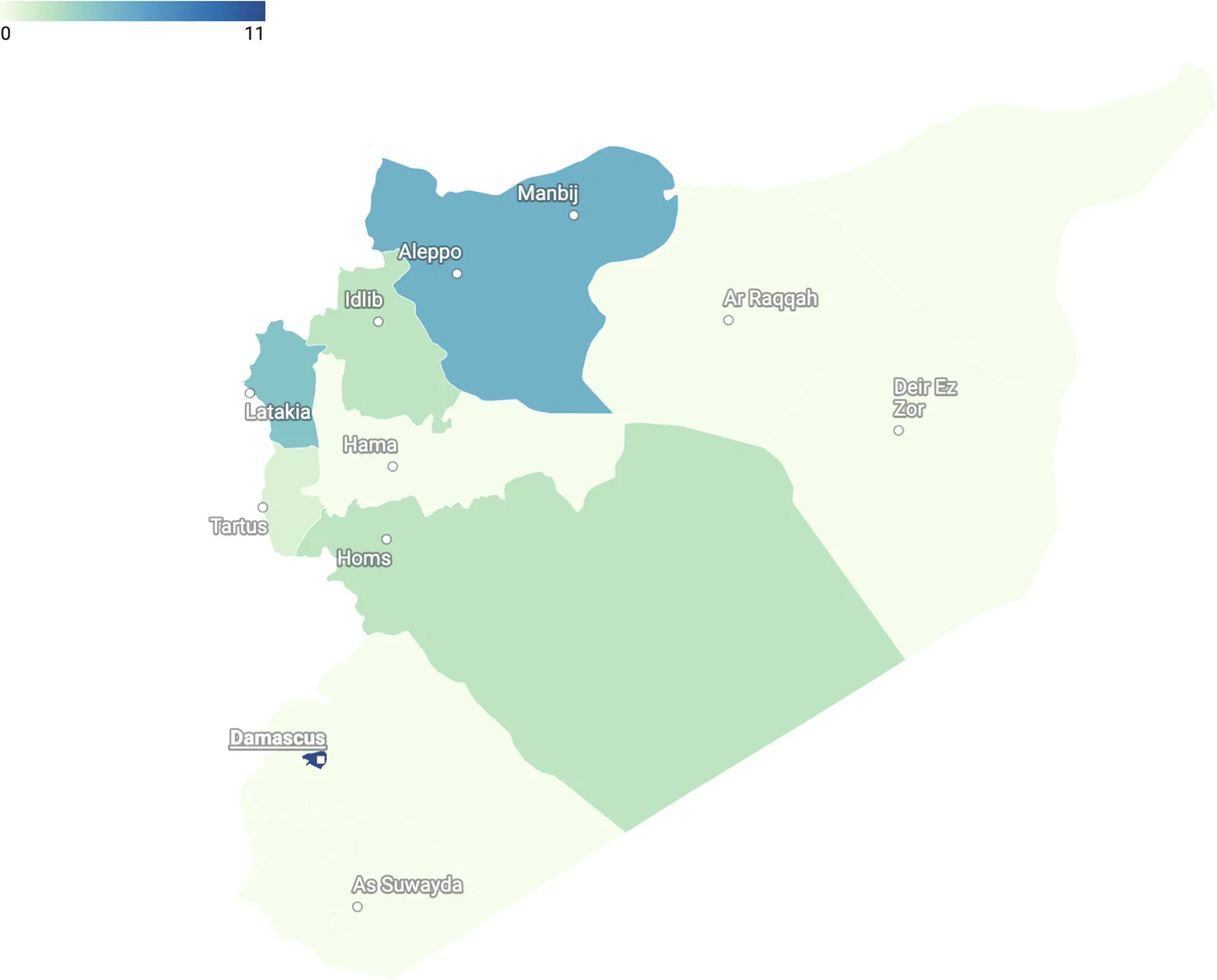
Geographic distribution of included studies across Syria. Map showing the geographic distribution of included studies by governorate. Color intensity reflects the number of included studies from each location, with darker shading indicating a greater number of studies. Figure made using Datawrapper.

The literature was geographically concentrated in a small number of cities. Damascus was the most frequently represented city (n = 11), followed by Aleppo (n = 5), Lattakia (n = 4), Idlib (n = 2), and Homs (n = 2); Tartous appeared in one multicity study (n = 1) (Figure 2). Most studies were conducted in hospital-based settings: inpatient-only populations accounted for the largest share (n = 12), followed by mixed inpatient/intensive care unit (ICU) cohorts (n = 3), outpatient-only cohorts (n = 2), ICU-only cohorts (n = 1), and mixed outpatient/inpatient cohorts (n = 1). Three studies did not clearly report the clinical setting (n = 3).

Specimen sources were heterogeneous, although urine, wound-related samples, and respiratory specimens predominated. Because many studies included more than one specimen type, these categories were not mutually exclusive. Wound/skin/pus/swab specimens were reported in 12 studies, urine in 11 studies, respiratory specimens, including sputum, bronchial washings, endotracheal aspirates, or other respiratory samples, in 10 studies, blood in 5 studies, and cerebrospinal fluid (CSF) in 3 studies. Three studies did not clearly report the specimen type. The distribution of organisms reported was as follows: *E. coli* (n = 15 studies), *P. aeruginosa* (n = 14), *K. pneumoniae or Klebsiella* spp. (n = 12), *Enterobacter* spp. (n = 8), *S. aureus* (n = 6), and *A. baumannii* (n = 4).

AST methods were most commonly based on disk diffusion. Kirby-Bauer or disk diffusion methods, including studies using cefoxitin disk diffusion for *S. aureus* /MRSA assessment, were reported in 18 studies. Broth microdilution was used in two studies, and one study reported using an E-test. Interpretive standards were inconsistently reported: Clinical Laboratory Standards Institute (CLSI) or National Committee for Clinical Laboratory Standards (NCCLS) guidance was explicitly mentioned in 12 studies, whereas 10 studies did not clearly specify whether CLSI, European Committee for Antibiotic Sucseptibility Testing (EUCAST), or another interpretive standard was used. Among the studies that reported using particular guidelines, the editions used varied.

### Summary resistance estimates

**Figure 3:**
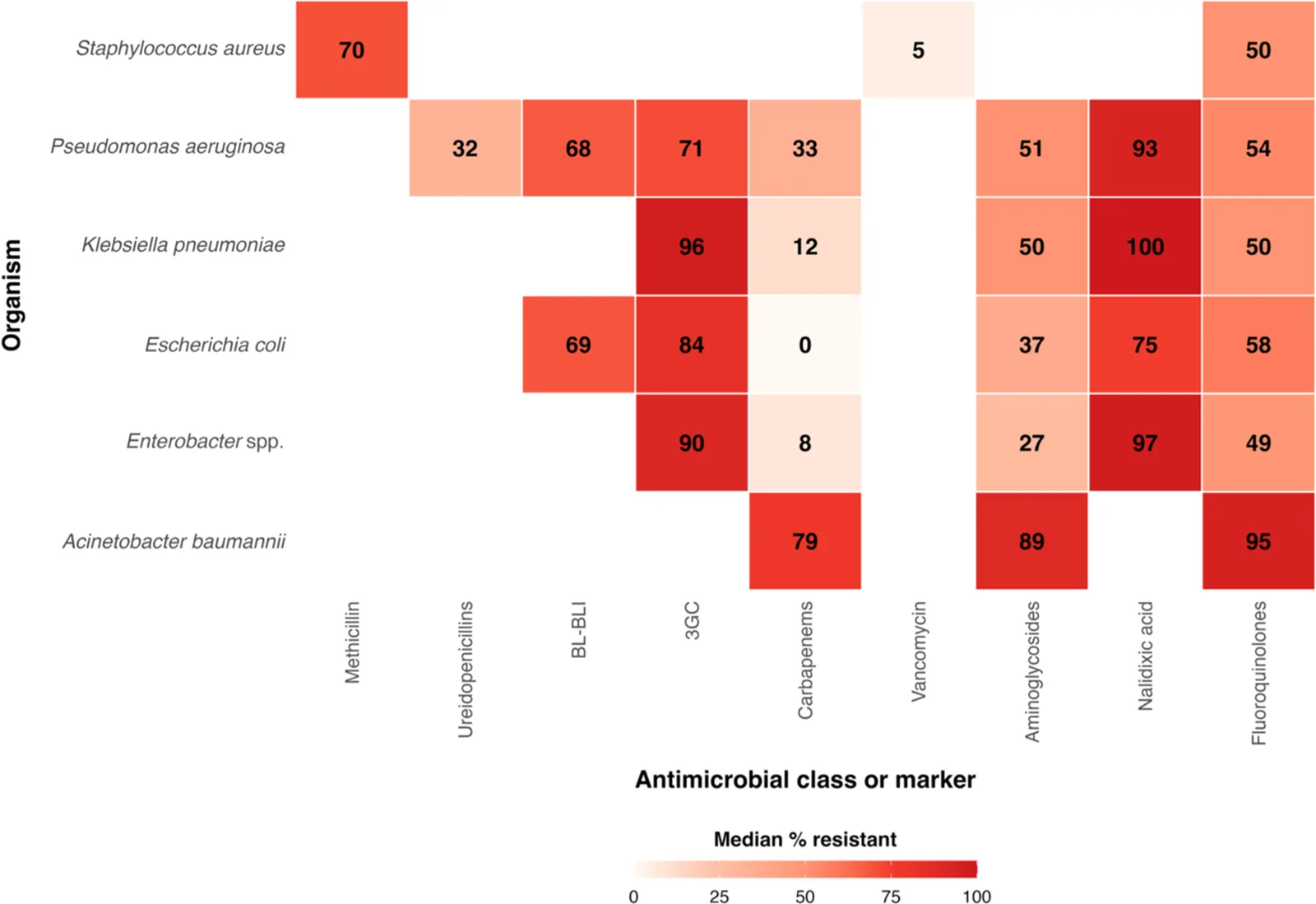
Heatmap of median antimicrobial resistance by organism and antimicrobial class/marker across included studies. Values represent the median percentage resistant across studies. Blank cells indicate combinations for which pooled summary data were not available. 3GC, third-generation cephalosporins; BL-BLI, β-lactam/β-lactamase inhibitor combinations.

Synthesized resistance estimates by organism and antibiotic class/marker are presented in Table 2 and Figure 3. Values are reported as the median study-level resistance percentage (IQR). Here, k denotes the number of contributing studies for each organism-class stratum. N denotes the pooled isolate total (sum of denominators across studies reporting sample sizes for that stratum). Antibiotic-level organism-agent estimates are provided in Table S4. A condensed cross-organism comparison is shown in Table S5, with a visual summary in Figure S1.

**Table 2:** Synthesized antimicrobial resistance by organism and antibiotic class/marker in Syria.

Organism	Antibiotic class/marker	Studies (n)	Total N (pooled)	Studies with N	Median resistance (IQR)
*A. baumannii*	Carbapenems	4	200	4/4	79.4% (51.3-91.7)
*A. baumannii*	Aminoglycosides	4	240	4/4	89.3% (84.9-95.1)
*A. baumannii*	Fluoroquinolones	4	219	4/4	94.7% (89.5-98.6)
*Enterobacter* spp.	third-generation cephalosporins	7	186	6/7	90.5% (56.3-93.5)
*Enterobacter* spp.	Carbapenems	7	241	6/7	8.2% (0.0-25.9)
*Enterobacter* spp.	Aminoglycosides	7	358	6/7	27.4% (11.3-57.1)
*Enterobacter* spp.	Nalidixic acid	2	42	2/2	97.0% (95.5-98.5)
*Enterobacter* spp.	Fluoroquinolones	7	308	6/7	48.7% (26.1-62.0)
*E. coli*	BL-BLI	2	890	2/2	69.2% (64.6-73.7)
*E. coli*	third-generation cephalosporins	10	299	7/10	83.6% (71.2-91.6)
*E. coli*	Carbapenems	6	810	4/6	0.0% (0.0-15.2)
*E. coli*	Aminoglycosides	10	1096	7/10	37.2% (12.9-58.4)
*E. coli*	Nalidixic acid	3	12	1/3	75.0% (74.7-81.5)
*E. coli*	Fluoroquinolones	10	1028	7/10	57.6% (42.1-70.7)
*K. pneumoniae*	third-generation cephalosporins	8	257	8/8	96.3% (80.5-100.0)
*K. pneumoniae*	Carbapenems	8	355	8/8	11.8% (0.0-71.5)
*K. pneumoniae*	Aminoglycosides	7	500	6/7	50.0% (37.8-75.4)
*K. pneumoniae*	Nalidixic acid	1	37	1/1	100.0% (100.0-100.0)
*K. pneumoniae*	Fluoroquinolones	7	429	7/7	50.0% (31.7-63.2)
*P. aeruginosa*	Ureidopenicillins	2	5	1/2	31.8% (15.9-47.7)
*P. aeruginosa*	BL-BLI	8	306	6/8	67.8% (41.1-100.0)
*P. aeruginosa*	third-generation cephalosporins	9	130	7/9	71.4% (60.4-94.1)
*P. aeruginosa*	Carbapenems	13	391	9/13	33.0% (0.0-47.1)
*P. aeruginosa*	Aminoglycosides	13	547	9/13	50.7% (45.0-68.8)
*P. aeruginosa*	Nalidixic acid	4	105	3/4	92.6% (84.7-100.0)
*P. aeruginosa*	Fluoroquinolones	14	540	10/14	54.0% (40.1-67.7)
*S. aureus*	Methicillin	2	112	2/2	70.5% (68.2-72.8)
*S. aureus*	Vancomycin	4	72	2/4	4.8% (1.5-14.0)
*S. aureus*	Fluoroquinolones	6	432	5/6	50.5% (41.4-51.8)

Values are presented as the median study-level percentage of resistant isolates (IQR) across included studies, stratified by organism and antibiotic class/marker. k denotes the number of contributing studies for each organism-class stratum. Total N (pooled) represents the sum of reported denominators across studies contributing to that stratum (based on study-level denominators within each class), and Studies with N indicates the proportion of contributing studies that reported denominators.

Abbreviations: *A. baumannii, Acinetobacter baumannii*; BL-BLI, β-lactam/β-lactamase inhibitor; *E. coli, Escherichia coli*; IQR, interquartile range; *K. pneumoniae, Klebsiella pneumoniae; P. aeruginosa, Pseudomonas aeruginosa; S. aureus, Staphylococcus aureus*.

Resistance to third-generation cephalosporins was consistently high across Gram-negative organisms, with the highest median resistance in *K. pneumoniae* (96.3%, IQR 80.5-100.0; k = 8; N = 257), followed by *Enterobacter* spp. (90.5%, IQR 56.3-93.5; k = 7; N = 186), *E. coli* (83.6%, IQR 71.2-91.6; k = 10; N = 299) and *P. aeruginosa* (71.4%, IQR 60.4-94.1; k = 9; N = 130).

For aminoglycosides, median resistance was highest in *A. baumannii* (89.3%, IQR 84.9-95.1; k = 4; N = 240). Resistance was lower in *P. aeruginosa* (50.7%, IQR 45.0-68.8; k = 13; N = 547) and *K. pneumoniae* (50.0%, IQR 37.8-75.4; k = 7; N = 500), and lower still in *E. coli* (37.2%, IQR 12.9-58.4; k = 10; N = 1096) and *Enterobacter* spp. (27.4%, IQR 11.3-57.1; k = 7; N = 358).

For carbapenems, resistance remained highest in *A. baumannii* (79.4%, IQR 51.3-91.7; k = 4; N = 200). Median resistance was lower in *P. aeruginosa* (33.0%, IQR 0.0-47.1; k = 13; N = 391) and *K. pneumoniae* (11.8%, IQR 0.0-71.5; k = 8; N = 355), lower in *Enterobacter* spp. (8.2%, IQR 0.0-25.9; k = 7; N = 241), and lowest in *E. coli* (0.0%, IQR 0.0-15.2; k = 6; N = 810).

For fluoroquinolones, median resistance was again highest in *A. baumannii* (94.7%, IQR 89.5-98.6; k = 4; N = 219). Resistance was also high in *E. coli* (57.6%, IQR 42.1-70.7; k = 10; N = 1028), *P. aeruginosa* (54.0%, IQR 40.1-67.7; k = 14; N = 540), *K. pneumoniae* (50.0%, IQR 31.7-63.2; k = 7; N = 429), *S. aureus* (50.5%, IQR 41.4-51.8; k = 6; N = 432), and *Enterobacter* spp. (48.7%, IQR 26.1-62.0; k = 7; N = 308).

For nalidixic acid, resistance was uniformly high where reported, including *K. pneumoniae* (100.0%; k = 1; N = 37), *Enterobacter* spp. (97.0%, IQR 95.5-98.5; k = 2; N = 42), *P. aeruginosa* (92.6%, IQR 84.7-100.0; k = 4; N = 105), and *E. coli* (75.0%, IQR 74.7-81.5; k = 3; N = 12).

Additional organism-specific estimates were available for selected classes. Among *P. aeruginosa*, resistance was 31.8% (IQR 15.9-47.7; k = 2; N = 5) for ureidopenicillins and 67.8% (IQR 41.1-100.0; k = 8; N = 306) for piperacillin-tazobactam. For *E. coli*, amoxicillin-clavulanate resistance was 69.2% (IQR 64.6-73.7; k = 2; N = 890). For *S. aureus*, methicillin resistance was 70.5% (IQR 68.2-72.8; k = 2; N = 112), while vancomycin resistance remained low (4.8%, IQR 1.5-14.0; k = 4; N = 72).

### Quality assessment

Using the NHLBI cohort/cross-sectional quality assessment tool, all included studies were rated as being of fair quality, with none achieving a rating of good quality (Table S6). Most studies clearly stated their objectives and defined the study population, and outcome measurement was generally described. However, participation/eligibility denominators were rarely reported, sample size justifications were uncommon, and multivariable adjustment for confounding was infrequently performed in studies evaluating predictors or between-group comparisons.

## Discussion

In this systematic review on AMR data from Syria, we evaluated resistance patterns across 22 studies spanning 2015-2025 and encompassing 6,367 isolates. First, third-generation cephalosporin resistance was uniformly high across Gram-negative organisms, with median resistance exceeding 70% for *P. aeruginosa* and approaching or exceeding 90% for *K. pneumoniae, E. coli*, and *Enterobacter* spp. Second, carbapenem resistance showed a bifurcated pattern: it was high in *A. baumannii* (median 79.4%, IQR 51.3-91.7), but notably lower in *K. pneumoniae* and *Enterobacter* spp., and lowest in *E. coli* (median, near zero). Third, fluoroquinolone resistance was consistently high across both Gram-negative and Gram-positive pathogens, with medians roughly around 50-60% for *E. coli, K. pneumoniae, P. aeruginosa,* and *S. aureus,* and >90% in *A. baumannii.*

We note the sparse available published data and that most publications originated from Damascus and Aleppo governorates, primarily from the cities rather than rural areas. Collectively, these findings reinforce the importance of strengthening surveillance for WHO BPPL organisms and improving access to timely, high-quality microbiology so that clinicians are better supported in making appropriate antibiotic choices for their patients [33]. The latter is of particular concern in the ICU setting. Given the inadequacy of microbiology services across Syria (both in quality and availability, particularly in the public sector), we must also interpret the available results with caution, as they may represent skewed resistance patterns; this may lead to an overestimation of resistance if samples are only sent when patients fail to improve on initial, empiric antimicrobials or an underestimation of the true resistance [34].

### Clinical implications

These findings indicate that in Syrian inpatient settings, where most evidence originates, empiric regimens anchored on third-generation cephalosporins may not provide reliable Gram-negative coverage. The high resistance rates to third-generation cephalosporins across *K. pneumoniae, E. coli*, and other *Enterobacter* spp. are consistent with previous literature reporting widespread ESBL-associated resistance in Syria [35]. This suggests that empiric ceftriaxone- or cefotaxime-based strategies are inadequate for particularly vulnerable patients with severe infections. Similarly, the consistently high fluoroquinolone resistance across both Gram-negative organisms and *S. aureus* argues against routine reliance on ciprofloxacin as an empiric “default,” especially for complicated infections or hospitalized patients. It emphasizes the importance of local antibiograms to support antimicrobial stewardship programs and empiric antibiotic choices for inpatients. However, given the limited data on community microbiology resistance patterns across most governorates in Syria, this finding cannot be extrapolated when considering empiric antibiotic guidelines for various community-acquired infections [34].

Carbapenem resistance among Gram-negative pathogens was variable compared with reports from the Eastern Mediterraneal Regional Office region [36]. Carbapenem-resistant *A. baumannii* (CRAB) resistance was high (median, 79.4%; IQR 51.3-91.7) and broadly consistent with the high CRAB burden reported in the region. Moghnieh et al. reported CRAB percentages of more than 65% overall and 83.3% in fragile or conflict-affected countries in the region [33]. A review of available data on *Acinetobacter* spp. isolates from Lebanon, Jordan, and Iraq from articles published between January 2015 and August 2020 identified CRAB percentages between 55% and 100%, with most reporting rates above 88%, overlapping with the upper range of our estimates [37]. Included in Moghnieh et al.’s review is a study from multiple sites in Tripoli, Lebanon (conducted from 2011 to 2013), which noted that CRAB rates among Syrian refugees (74%) were higher than those among Lebanese patients (47%) [38]. The disproportionately high carbapenem resistance in *A. baumannii* may reflect its predominance in health care settings, particularly ICUs, where prolonged hospitalization, broad-spectrum antibiotic exposure, and nosocomial transmission favor resistant strains. These pressures may be amplified in Syria by conflict-related disruption of health care infrastructure, infection prevention and control, and antimicrobial stewardship. Additionally, clonal CRAB outbreaks could disproportionately influence resistance estimates; however, the included studies lacked sufficient strain-typing data to distinguish sporadic from outbreak-associated isolates. This pattern underscores the importance of early effective therapy for high-risk patients and the imperative to apply carbapenem-sparing strategies to limit emerging resistance through stewardship. In practice, the results support a strategy in which escalation to carbapenems is guided by syndrome severity, local resistance patterns, and susceptibility testing, with early de-escalation when susceptibilities permit. However, aminoglycoside resistance was also substantial among *E. coli* at 37.2% (IQR 12.9-58.4), *K. pneumoniae* at 50.0% (IQR 37.8-75.4), and *Enterobacter* spp. at 27.4% (IQR 11.3-57.1).

Carbapenem resistance in *Enterobacter* spp. and *E. coli* was lower, at 8.2% (IQR 0.0-25.9) and 0.0% (IQR 0.0-15.2), respectively. For *K. pneumoniae*, carbapenem resistance was 11.8% (IQR 0.0-71.5). For *P. aeruginosa,* the combination of moderate-to-high resistance across multiple classes, including carbapenems and fluoroquinolones, suggests that empiric anti-pseudomonal coverage, when clinically indicated, should be informed by facility antibiograms and prioritized for rapid susceptibility-directed narrowing; this is particularly relevant in the ICU setting.

Although fewer studies addressed Gram-positive resistance, the available data suggest a significant MRSA burden, with a median methicillin resistance of 70.5%. These findings underscore the importance of considering local MRSA epidemiology when selecting empiric therapy for serious *S. aureus* infections. Reported vancomycin resistance was low (median 4.8%); however, if confirmed, even this level of resistance would be clinically concerning given that confirmed vancomycin-resistant *S. aureus* (VRSA) remains rare. Nevertheless, these findings should be interpreted cautiously. Variability in microbiological methods and susceptibility testing raises the possibility that some reported vancomycin-resistant isolates may not represent confirmed cases of VRSA.

### Study quality and strength of evidence

All included studies were rated as fair quality using the NHLBI observational cohort/cross-sectional tool. This rating reflects recurring constraints in the underlying literature. These findings are in line with a 2020 systematic review of AMR in the Middle East that found that studies showed heterogeneity in study design, laboratory methods, and standards for interpretation of results, and an overall high risk of bias [39]. There is a paucity of rigorous data both before and since the onset of conflict in March 2011 to adequately contextualize the burden of AMR in Syria [7].

Among the data available, the evidence base was concentrated in a limited number of governorates. Consequently, the synthesized estimates likely reflect resistance ecology in a subset of referral centers and larger hospitals rather than nationwide patterns. Additionally, this concentration limits confidence in regional comparisons. Nonetheless, the internal consistency of several high-level signals, especially high third-generation cephalosporin resistance among major Gram-negative organisms and very high resistance in *A. baumannii,* suggests that the overall direction of findings is robust across multiple institutions and time periods.

Our synthesis approach was selected to align with these realities. Because included studies varied in tested antibiotic panels, specimen types, and settings, we summarized resistance at the study level within antibiotic classes and aggregated across studies using medians and IQRs, ensuring that each study contributed one estimate per organism-class combination. This approach is robust to outliers and avoids disproportionately weighting studies that tested a larger number of agents within a class. Nevertheless, the strength of inference varies by the number of contributing studies, and estimates based on few studies (e.g., nalidixic acid or selected anti-pseudomonal classes) should be interpreted as less stable.

### Policy implications

The findings highlight the need for pragmatic, scalable improvements in AMR surveillance and antibiotic stewardship in Syria. The literature consistently demonstrates that even in fragile health systems, sustained improvements are achievable through standardized methods, multicenter collaboration, targeted capacity building, and strong governmental support, although contextualization to local realities remains essential [40,41]. In Syria, these priorities align with the Ministry of Health’s ongoing efforts to renew the national AMR response, strengthen microbiology services, and integrate AMR surveillance into broader public health systems. At the surveillance level, the available evidence would be strengthened by harmonized reporting methods, including consistent definitions for community- versus hospital-acquired isolates, explicit duplicate-isolate policies such as using the first isolate per patient within a defined interval, standardized organism-specific antibiotic panels, and clear documentation of interpretive standards, including the CLSI or EUCAST version and quality-control guidelines.

A practical next step would be to move from fragmented laboratory reports toward a more unified national surveillance model that can contribute to the WHO Global Antimicrobial Resistance and Use Surveillance System (GLASS). This could include incorporating AMR data into existing public health information systems and linking microbiologic data with clinical syndromic surveillance platforms, such as the Early Warning, Alert and Response System, particularly for priority pathogens and high-risk syndromes. Multicenter sentinel surveillance networks connecting public hospitals, private laboratories, and high-volume facilities would help generate comparable longitudinal antibiograms and support periodic updates to empiric treatment guidelines. Such a model would also allow surveillance efforts to begin in selected high-capacity sites while gradually expanding geographic and sectoral coverage.

A recent Knowledge, Attitudes, and Practices survey of AMR among 1179 health care professionals suggested that few clinicians had access to local antibiotic policies, with many relying on international or generic guidelines that may not reflect Syrian resistance patterns [4]. Local or national empiric antibiotic guidelines based on Syrian antibiogram data are therefore essential. These should be established alongside broader AMR control measures, including strengthened infection prevention and control measures, antimicrobial stewardship committees, and routine feedback of facility-level resistance data to clinicians. Finally, the relative scarcity of outpatient and community-level studies represents a strategic blind spot. Expanding surveillance beyond hospital-based datasets to ambulatory settings, uncomplicated and complicated infection syndromes, and private-sector laboratories would better inform empiric therapy across the full continuum of care.

### Limitations

This review is limited by the structure and reporting quality of the available evidence. Most studies were single-center and hospital-based, with uneven geographic coverage, limiting national representativeness and regional comparisons. Laboratory methods and reporting varied, and denominators, duplicate-isolate handling, and AST interpretive standards were inconsistently documented. Inconsistent organism-antibiotic denominator reporting precluded denominator-weighted pooled estimates and limited our ability to account for differences in the number of isolates tested for each agent within a class. Our median-based approach minimized the influence of heterogeneous antibiotic panels and extreme values but may obscure within-class variation or underrepresent high resistance to selected agents; conversely, using the highest reported value could overestimate resistance when based on small denominators or outlier studies. Some organism-antibiotic strata were also supported by few studies, reducing precision. Nevertheless, this review provides a valuable assessment of the current landscape of AMR data in Syria.

## Conclusion

Available data, though sparse and geographically uneven, suggest high levels of resistance, particularly among WHO priority pathogens. Syria is in the process of renewing its AMR National Action Plan, the core pillars of which are strengthening microbiology services, establishing national and local Antimicrobial Stewardship programs, and contributing to international surveillance programs, including GLASS, as part of integrated disease surveillance. Data from this review provide a starting point to support these measures, including the development of national antimicrobial guidelines.

## CRediT authorship contribution statement

**Bayan Galal:** Writing – review & editing, Writing – original draft, Software, Formal analysis, Data curation, Conceptualization. **Nadia Barouk:** Data curation, Conceptualization. **Ahmad Kejah:** Writing – review & editing, Conceptualization. **Marwan Osman:** Writing – review & editing, Conceptualization. **Nabil Karah:** Writing – review & editing, Conceptualization. **Salam Abbara:** Writing – review & editing, Conceptualization. **Yasir Farrouh:** Writing – review & editing, Writing – original draft, Conceptualization. **Shaffi Fazaludeen Koya:** Writing – review & editing, Conceptualization. **Antoine Abou Fayad:** Writing – review & editing, Conceptualization. **Souha S. Kanj:** Writing – review & editing, Conceptualization. **Ahmad Darwish:** Writing – review & editing, Conceptualization. **Omar Dewachi:** Writing – review & editing, Conceptualization. **Aula Abbara:** Writing – review & editing, Writing – original draft, Supervision, Data curation, Conceptualization.

## Declaration of competing interest

The authors have no competing interests to declare.

## Appendix Supplementary materials

Image, application 1
